# Non-invasive Hyperglycemia Detection via Electrocardiogram Using Discrete Wavelet Transform and Machine Learning

**DOI:** 10.7759/cureus.80548

**Published:** 2025-03-13

**Authors:** Oscar I Coronado-Reyes, Adriana C Téllez-Anguiano, Luis A Castro-Pimentel, Jose A Gutierrez-Gnecchi

**Affiliations:** 1 Graduate Studies and Research Division, TecNM Instituto Tecnológico de Morelia, Morelia, MEX

**Keywords:** diabetes mellitus, discrete wavelet transform, heart rate variability, k-nearest neighbors, support vector machine

## Abstract

Introduction and aim: Diabetes mellitus (DM) is a chronic metabolic disorder characterized by elevated blood glucose levels (BGLs), traditionally monitored through invasive methods. Continuous monitoring of BGL is essential to prevent severe complications. This study proposes a non-invasive approach to detect hyperglycemia by analyzing electrocardiogram (ECG) signals using the discrete wavelet transform (DWT) and machine learning techniques. The relationship between diabetes and electrocardiographic (ECG) signals is highly relevant in biomedicine, as DM is a significant risk factor for cardiovascular diseases.

Materials and methods: A total of 210 individuals (healthy and diabetic, aged 18-70 years) were analyzed, with ECG signals processed using DWT to extract heart rate (HR) in beats per minute (bpm) and heart rate variability (HRV) parameters. These features were used as inputs for support vector machine (SVM) and k-nearest neighbors (KNN) classifiers to distinguish between normal and hyperglycemic states (>150 mg/dL).

Results: The DWT-based automatic feature detection achieved an accuracy of 99.8% for both HR and HRV. Pearson correlation analysis revealed moderate correlations between glucose levels and HR (0.2985) and HRV (-0.373), with a combined correlation index of 0.6428.

Conclusion: Classification results showed an accuracy of 97% for normal glucose levels and 93% for hyperglycemia detection using both SVM and KNN. These findings indicate that ECG signal characteristics can serve as an adjunct for non-invasive hyperglycemia detection.

## Introduction

Diabetes mellitus (DM) is a chronic metabolic disease characterized by elevated blood glucose levels (BGLs). DM arises from insufficient insulin production or ineffective insulin utilization [1,2].

DM is a global health problem affecting approximately 50 million people, with type 2 diabetes comprising 90-95% of cases [3]. Clinically accepted BGL measurement methods are invasive techniques that require blood sampling and calculation of BGL through the chemical reaction between the test reactive and the blood, causing discomfort and potential infection risks [4,5].

To address these limitations, recent research has explored non-invasive BGL measurement techniques, such as near-infrared spectroscopy (NIR), optical methods, and Raman spectroscopy [6-9]. While promising, these methods are limited due to physiological variables that can affect the measurement. These techniques demonstrate good results within normal ranges (70-150 mg/dL), but errors tend to increase in hyperglycemic ranges.

Electrocardiographic (ECG) signals can offer an alternative for BGL estimation or detecting hyperglycemic periods, as glucose levels influence the cardiovascular system and autonomic nervous activity [10-16]. Unlike optical techniques, ECG is less affected by external factors, does not require complex hardware, and can be easily integrated into wearable devices.

ECG records the heart's electrical activity generated by the depolarization and repolarization of the atria and ventricles. Its main components are the P wave, QRS complex, and T wave. The connection between DM and ECG signals is crucial in biomedicine, as ECG-based monitoring can help in the early detection of cardiac dysfunction. 

Glucose fluctuations influence heart rate (HR) and heart rate variability (HRV), making ECG a viable tool for continuous non-invasive hyperglycemia detection in real-world settings. However, existing studies lack robust models for glucose monitoring using ECG signals. Our methodology aims to provide a reliable, non-invasive tool for hyperglycemia detection by analyzing ECG features using machine learning models.

## Materials and methods

Participants

This study involved 210 volunteers aged 18 to 70. Participants with known cardiovascular diseases and people who take medications associated with the nervous system were excluded. The inclusion criteria were smokers, caffeine consumers, and people who perform any physical activity. Demographic classifications such as gender, age, and BMI were not considered. The studies focus on people with DM type 2.

ECG records were acquired using lead VII, and blood glucose was measured using the Accu-Chek Performa® glucometer (Roche Diabetes Care, Switzerland). The study was conducted at the TecNM Morelia Technological Institute, Mexico, in the period from June 2023 to October 2024, and it was approved by the Institutional Ethics Committee of the Morelia Technological Institute with approval number 001/2025; it is important to mention that the criteria established in the Declaration of Helsinki were followed in the study.

Sampling

**Figure 1:**
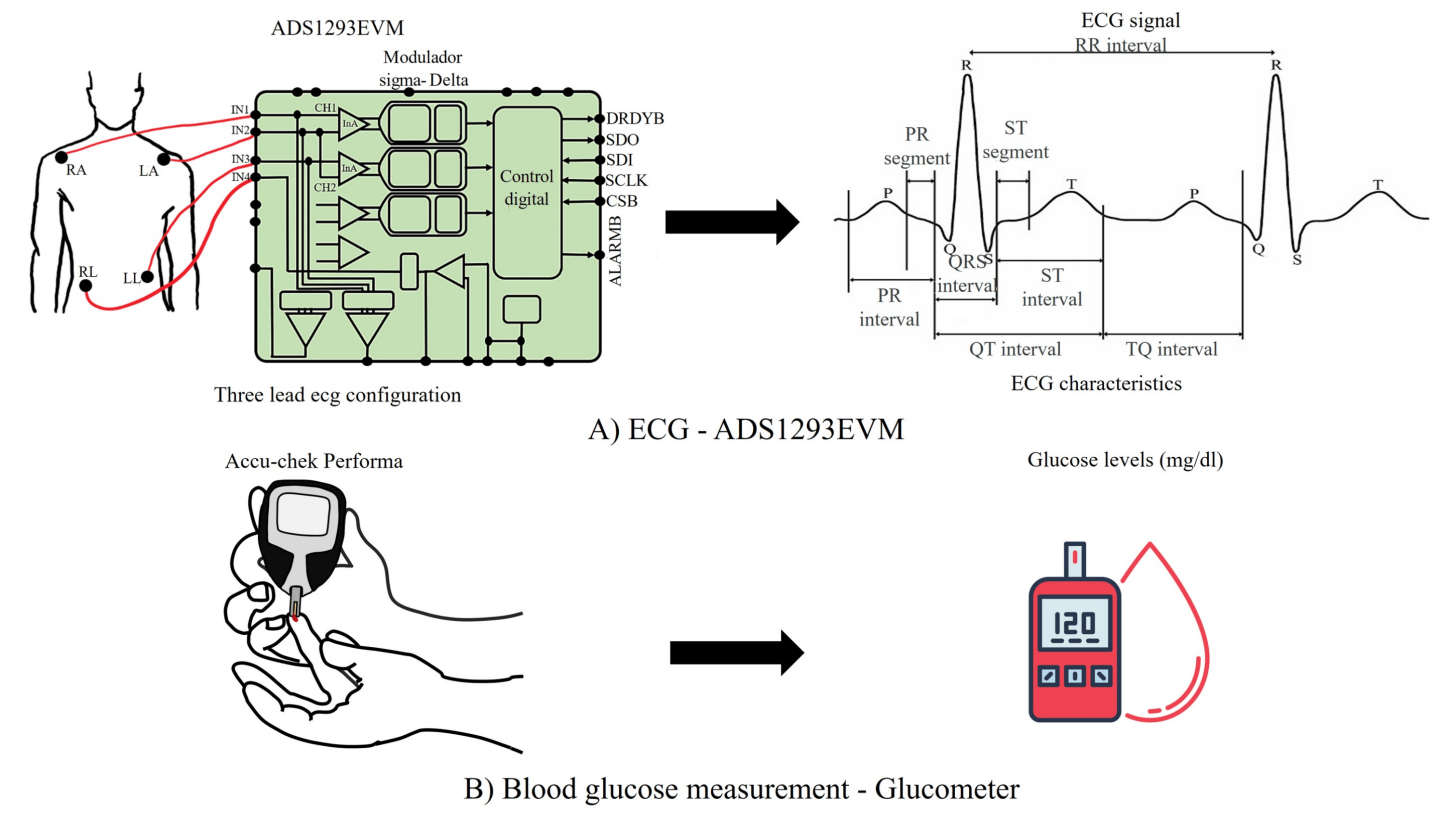
ECG and blood glucose measurement protocols: A) ECG measurement using the ADS1293EVM; B) blood glucose measurement using a commercial glucometer. Credit: Image created by the authors

To measure the ECG signals, the ADS1293EVM evaluation card was used with the following characteristics: 24-bit resolution, three-lead configuration, sampling frequency Fs = 1.4 kHz, isolation security and protection in power supplies, data import for post-processing, and free software for data management and acquisition (Figure 1A).

The ADS1293EVM allows measurements of leads VI, VII, and VIII; lead VII is selected because changes in glucose levels, particularly hyperglycemia, can affect the autonomic nervous system, leading to alterations in HR and HRV; lead VII is sensitive to these changes because it captures electrical activity influenced by both sympathetic and parasympathetic inputs.

The ECG signals were sampled at 1.4 kHz to capture high-frequency components that may be relevant for detecting subtle changes associated with hyperglycemia. While typical ECG sampling rates range between 250 Hz and 500 Hz, the higher rate was chosen to ensure sufficient resolution for detailed analysis of HR and HRV.

BGL was measured using a commercially available device, the Accu-Chek Performa®, validated according to ISO 15197:2013 standards (Figure 1B). To use the device, the calibration and use techniques of the device available in the manufacturer's data sheet must be followed.

For proper sample collection, the candidate for ECG recording must have a minimum of one hour of inactivity, not have had caffeine intake, and not be in a stressful environment. The patient must lie down for five minutes to relax the heart, then the electrodes are connected as shown in Figure 1A, and then an eight-second ECG signal is recorded.

Once the ECG is captured, the BGL is measured with a maximum difference of five seconds. This is established as a protocol to prevent prolonged intervals between samples, with the samples being considered instantaneous.

The eight-second ECG recording was chosen to balance the need for sufficient data capture with practical constraints in clinical settings. While longer recordings are typically preferred for HRV analysis, our preliminary studies indicated that eight seconds provided adequate data for reliable feature extraction in this context.

Study procedure

The research begins by obtaining a database of 210 records (ECG and BGL) in a glucose range of 70 mg/dl to 500 mg/dl. Once the complete record of ECG signals is available, signal processing is carried out using MATLAB software to measure HRV and HR using the DWT and detection of maximums and minimums.

DWT was selected due to the good results shown in previous work for the detection of features. Due to the time-frequency analysis and multi-resolution analysis [17], Symlet 4 was chosen for its ability to capture both low- and high-frequency components relevant to HRV analysis. The 10-level decomposition was selected to ensure sufficient resolution for detecting subtle changes in ECG signals associated with hyperglycemia, without being affected by the noise.

ECG samples were taken in a noise-free, temperature-controlled space (temperature of approximately 23-26 degrees Celsius).

With the HRV and HR parameters detected, the Pearson correlation is used to determine their relationship with the BGL. Finally, HRV and HR are used as the inputs for two classification models, support vector machine (SVM) and k-nearest neighbor (KNN), to obtain two classes, normal glucose and hyperglycemia.

Feature extraction

The feature extraction module obtains the HR and HRV features, which serve as input to the classifiers. The DWT technique allows post-processing of the digital ECG signal, enabling baseline correction and noise removal while preserving signal wave characteristics, and separating the signal into high and low-frequency components [17].

The DWT allows the representation, decomposition, and reconstruction of signals that exhibit abrupt changes in their time-frequency components, employing multi-resolution analysis. Multi-resolution analysis uses variable-length windows adapted to the signal’s frequency change. DWT enables using large time intervals in segments requiring higher precision in low frequency while employing smaller intervals where high-frequency information is needed [18]. The continuous wavelet transform (CWT) is defined by equation (1):

$W_{f}\left( U,S \right)=f*\psi_{u,s}=\int_{-\alpha}^{\alpha}f\left( t \right)\frac{1}{\sqrt{s}}\psi\frac{t-u}{s}dt$ (1)

where f(t) is the continuous signal, Ψ is a wavelet function (mother wavelet), s represents the scaling factor of the wavelet function, that contracts if s < 1 and expands if s > 1, and u is the translation factor to shift the location of the wavelet function.

To apply the CWT to signals processed in digital systems such as microcontrollers, software, etc., it is necessary to discretize the CWT. The DWT of a digital signal is defined by equations (2) and (3):

$S_{2j}f\left( n \right)=\sum h_{k}S_{2j-1}f\left( n-2^{j-1}k \right)$ (2)

$W_{2j}f\left( n \right)=\sum g_{k}S_{2j-1}f\left( n-2^{j-1}k \right)$ (3)

where S_2j_f(n) are the approximation coefficients, W_2j_f(n) are the detail coefficients, 2j is the scale of the wavelet transform of f(n) and n is the number of samples. The terms h_k_ and g_k_ correspond to the coefficients of a low-pass and high-pass filter.

To perform the detection and feature extraction in ECG signals, an algorithm was developed in MATLAB software with the following steps:

1. Reading .txt file: This document contains amplitude and time data captured in an eight-second ECG recording with the ADS1293EVM, with Fs of 1400 Hz.

2. Decomposition: With the DWT technique, multi-resolution analysis was used to decompose the ECG signal into 10 levels, and to analyze the high- and low-frequency components, a Symlet 4 was used as the mother wavelet.

3. Baseline correction: In this step, the approximation component 10 was eliminated, which contains the lowest frequency elements corresponding to the movement caused by breathing or the moves of the subject at the time of taking the sample.

4. Noise removal: In this stage, the component containing the line noise (60 Hz) was removed.

5. Reconstruction of high-frequency components: In this stage, the highest-frequency components, which contain the information of the QRS complex, were reconstructed (details 7, 6, 5, and 4).

6. Local maxima: In this stage, local maxima were detected in the signal reconstructed with the high-frequency elements.

7. Global maximum: At this stage, the maximum in the reconstructed high-frequency signal was detected.

8. Peak and RR time: To detect R times and peaks in the eight-second record, the values of local maxima located in the reconstruction of high-frequency components were compared with a window in amplitude defined by the global maximum. The local maxima found within the window are identified as R peak. Once the R peak is detected, the corresponding time and amplitude are stored and located in the corrected and filtered signal.

9. RR interval: Once the R times have been identified, the differences in time of the consecutive R peaks are obtained.

10. HRV: To measure HRV, the standard deviation between the RR intervals found in the eight-second record is obtained.

11. HR: HR is measured by the time when each heartbeat occurs in the record.

**Figure 2:**
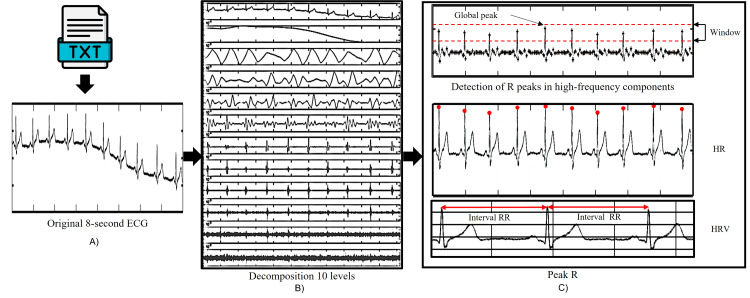
ECG signal processing stages: A) ECG signal acquisition; B) ECG signal decomposition using DWT; C) ECG signal feature detection and extraction. DWT: discrete wavelet transform Credit: Image created by the authors

Figure 2 shows the descriptive diagram of the steps mentioned for the HR and HRV automatic detection.

Statistical analysis

The most used sample estimator to evaluate the linear association between two variables, X and Y, is the Pearson correlation coefficient shown in equation (4). This index measures whether the points tend to lie in a straight line. It can take values between -1 and +1. It is a parametric statistical method since it utilizes the mean and the variance and therefore requires normality assumptions for the analyzed variables [19].

$r=\frac{S_{xy}}{S_{x}S_{y}}$ (4)

where r is the Pearson correlation coefficient, S_xy_ is the sample covariance between x and y, S_x_ is the variance of x, and S_y_ is the variance of y. The magnitude of r considers the relationship between variables; a correlation is low below 0.30 in absolute value, a moderate association between 0.30 and 0.70, and a high above 0.70 [19].

Pearson correlation analysis was conducted to assess the relationship between ECG features and BGL. HR and HRV were used as input variables for SVM and KNN classifiers, with data split into 60% training and 40% validation.

Automatic classification

Automatic classification consists of determining the class to which an element belongs based on its characteristic features; these characteristics are used as inputs. Automatic classification begins with collecting data from a specific population. The collected data is divided into two subsets: training and validation. The training data used is used to develop the classifier model, while the validation data is used as a reference to validate the efficiency of the designed model. The classifier models are KNN and SVM.

**Figure 3:**
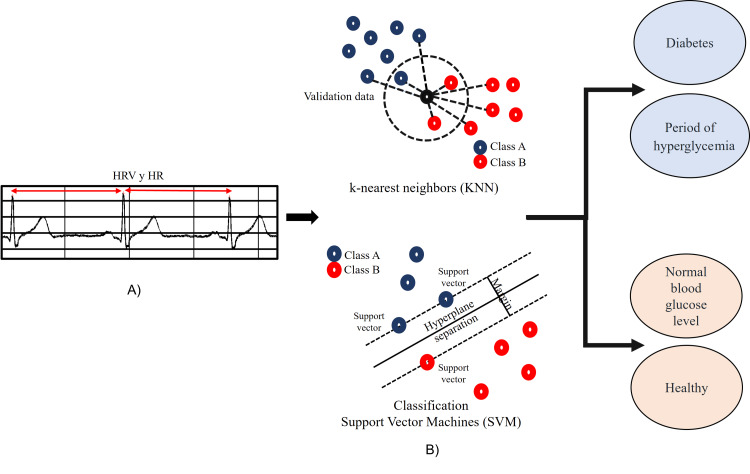
Blood glucose level classification using A) ECG features, B) SVM and KNN classifiers. SVM: support vector machine; KNN: k-nearest neighbor; HR: heart rate; HRV: heart rate variability Credit: Image created by the authors

The classification techniques were implemented to classify healthy people and people with DM, normal glucose (70 mg/dl-150 mg/dl), and periods of hyperglycemia (greater than 150 mg/dl) based on the characteristics (inputs). The KNN method (Figure 3) is based on finding the shortest distance between the data to be evaluated (inputs) and the KNN in the training data. The first step is to find the Euclidean distance in the training data and then the number of k neighbors to be used in the classification.

Finally, the classification is performed using the k Euclidean distances between the training data and the sample to be classified using equation (5) [20].

$dis\left( x,y \right)=\sqrt{\sum_{i=0}^{n} \left( y_{i}-x_{i} \right)^{2}}$ (5)

where x is the training data, y is the validation data, and n is the dimension of the data.

The SVM (Figure 3) is a supervised learning algorithm used in many classification and regression problems, including medical signal processing applications, natural language processing, and image recognition.

The goal of the SVM algorithm is to find a hyperplane that separates two or more different classes of data in the best possible way; it implies the hyperplane with the widest margin between the two classes. Support vectors refer to a subset of the training observations that identify the location of the separating hyperplane. The standard SVM algorithm is formulated for binary classification problems [21].

## Results

Feature detection

Following the steps mentioned in the feature extraction stage, the 210 samples were analyzed, obtaining the results shown in Table 1. These results correspond to an excerpt of the 210 samples, where the values of HR and HRV obtained with the DWT can be observed.

**Table 1:** Extract (11 samples) of glucose level measurement and HR and HRV features detection results by the DWT. HR: heart rate; HRV: heart rate variability; DWT: discrete wavelet transform; bpm: beats per minute

Sample	Glucose level (ml/dl)	HR (bpm)	HRV
1	90	71.33	0.044035
2	109	71.76	0.024101
3	101	74.278	0.02682
4	151	82.799	0.009005
5	80	53.881	0.057756
6	162	68.265	0.018142
7	116	64.043	0.04764
8	500	101.62	0.0042798
9	182	85.505	0.0125568
10	291	73.77	0.0068605
11	183	60.69	0.017971

As can be seen in the results, HRV is significantly reduced in individuals with diabetes, primarily due to the negative effects of chronic hyperglycemia on autonomic nervous system regulation. In contrast, healthy individuals exhibit higher HRV levels, indicating a more robust and balanced cardiac autonomic function, reflecting overall better cardiovascular health.

With the HRV analysis, it was found that people with BGL between 70 mg/dl and 150 mg/dl had a variability in the range of 0.02 to 0.2, people with BGL between 150 mg/dl and 200 mg/dl had a variability in the range of 0.019 to 0.009, while people with BGL had a variability less than 0.009.

Correlation

To know the effect caused by diabetes and glucose levels on the ECG, Pearson correlations were obtained (Table 2) between the characteristics of interest (HR and HRV) and the BGL.

**Table 2:** Pearson correlation between the blood glucose level and the ECG signal features. HR: heart rate; HRV: heart rate variability

Correlation	HR	HRV	HR/HRV
Blood glucose level	0.2985	-0.373	0.6428

According to the results presented in Table 2, the correlation between HR and blood glucose is moderate, the correlation between HRV and blood glucose is negatively moderate, and the correlation between blood glucose and the HR/HRV factor is high.

Based on the results provided in Table 2, it can be mentioned that the correlation of BGL with HR and HRV is moderate and high. Therefore, these features are used as inputs to classifiers to detect periods of hyperglycemia.

Classification

**Figure 4:**
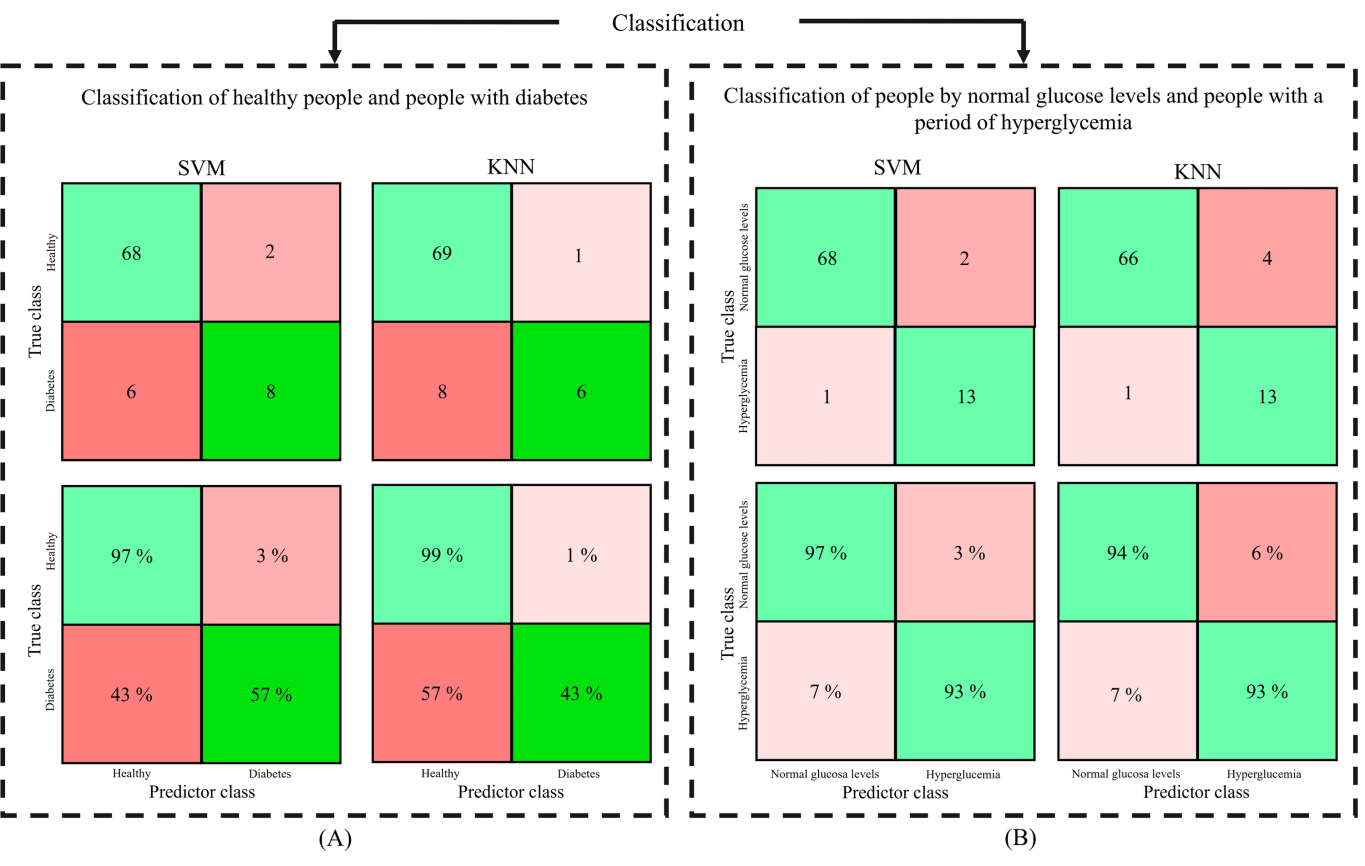
SVM and KNN classification results concerning A) healthy and diabetic people and B) normal and high glucose levels. SVM: support vector machine; KNN: k-nearest neighbor

With the factors of interest (HR and HRV), the SVM (using an RBF kernel and C = 1) and KNN (with k = 5) algorithms were evaluated, first to classify healthy individuals and those with DM (Figure 4A). The algorithms were designed using the 210 analyzed data, where 66.66% of the data (140 samples) was used for training and 33.33% (70 samples) for validation.

Figure 4 presents the classification results in confusion matrices. In each matrix, the top-left box indicates the number of true positives (TP), which are the correct predictions of the positive class. The bottom-left box shows the false positives (FP), representing instances of the negative class incorrectly classified as positive. The top-right box corresponds to the false negatives (FN), which are actual positive cases mistakenly classified as negative. Finally, the bottom-right box displays the true negatives (TN), representing instances of the negative class accurately identified. The upper confusion matrix presents the results considering the number of samples, the lower shows their corresponding percentages.

As observed in Figure 4A, the lowest percentages in the classification are in the classification of people with DM, having a 57% classification accuracy using the SVM method. The accuracy percentage in the classification with the KNN technique decreases to 43%. The accuracy percentage in healthy individuals increases, reaching 97% on SVM to 99% in the KNN technique. The classification errors occur because there are eight samples with abnormal glucose levels (levels higher than 150 mg/dl) in the database, which were taken from individuals considered healthy. These samples exhibit characteristics in the ECG like those of individuals with DM.

To know the effect caused by abnormal blood glucose values in the eight samples, the SVM and KNN algorithms were analyzed to classify normal GBL and abnormal GBL (hyperglycemia); the obtained results are shown in Figure 4B.

As observed in the classification of individuals with normal and hyperglycemic GBL, the accuracy percentages increase, reaching 97% in the detection of normal GBL and 93% in the classification of people with hyperglycemia, using SVM. On the other hand, using KNN, the accuracy percentages also increased, reaching 94% in the classification of individuals with normal GBL and 93% in people with hyperglycemia. The main errors in classification correspond to people with DM who have normal GBL (less than 150 mg/dl).

DWT-based feature extraction achieved 99.8% accuracy in detecting HR and HRV. Pearson correlation results showed moderate correlations between BGL and HR (0.2985) as well as HRV (-0.373). When both features were combined, a correlation index of 0.6428 was obtained. Classification accuracy reached 97% for normal glucose levels and 93% for hyperglycemia detection using SVM and KNN.

## Discussion

To validate the classifiers' performance, Table 3 presents a comparison between previous works and this proposal; class corresponds to the performed classification, where 1 indicates classification as DM, and 2 indicates classification as normal glucose levels and hyperglycemia.

**Table 3:** Comparison of the classification results between previous studies and this work. KNN: k-nearest neighbor; SVM: support vector machine; DM: diabetes mellitus

Authors	Methods	Class	Accuracy
[11]	Convolutional neural network and long short-term memory	1	95.1% DM
[15]	KNN	1	92.02% DM
	Decision tree	1	92.64% DM
[22]	Convolutional neural network followed by SVM	1	95.7% DM
[23]	Artificial neural network	1	96.2% DM
	SVM	1	95.2%
[24]	AdaBoost classifier	1	86% DM
[25]	Least square - SVM	1	95.63% DM
[26]	Logistic regression	1	92% DM
This work	SVM	1	97% healthy
			57% DM
		2	97% normal glucose levels
			93% hyperglycemia

As can be seen in Table 3, compared to former studies, the classification results between DM and healthy individuals exhibit a reduced accuracy primarily due to the database used; in previous studies, the database is formed by samples adequately classified in normal and DM. In this work, instead, the analysis is based on real-world measurements that include individuals with diabetes who have been misclassified as normal. This introduces classification errors, as the dataset inherently reflects the inaccuracies of clinical diagnostics. To address this limitation, the classification approach was refined to distinguish between individuals with normal glucose levels and those with hyperglycemia. This adjustment aligns directly with the measured values, increasing the classification performance, thereby mitigating the impact of diagnostic misclassifications and providing a more reliable basis for analysis.

Based on this study, it can be stated that with a database of 210 ECG signal records in glucose ranges of 70 mg/dl to 500 mg/dl, the classification percentages are up to 90% for detecting periods of hyperglycemia, with HR and HRV as main characteristics. The KNN and SVM methods had the same percentage of accuracy in the classification of people with episodes of hyperglycemia (93%); the difference occurred in the classification of normal BGL, with the SVM technique giving better results. It is important to mention that the main errors in the classifications are due to individuals with DM showing normal glucose levels below 150 mg/dl, but the characteristics in the ECG signal, mainly HRV, presented values similar to those found in elevated GBL.

On the other hand, it is important to mention that the ADS1293EVM acquisition card is a good evaluation platform to extract the characteristics of interest in the ECG signals (HR and HRV); its resolution and Fs allow identifying small changes related to elevated glucose levels. Also, the algorithms implemented by the DWT technique in automatic feature detection showed an efficiency of 99.7%; this provides reliable information on the measured parameters (HRV and HR).

In this study, a trend of change in HRV that affects individuals with diabetes and has glucose levels higher than normal was observed, which helped to achieve good classification results. Finally, it should be mentioned that glucose samples were taken without affecting the volunteers’ lifestyle (food intake, medications, exercise, etc.). This was done to measure BGL regardless of previous conditions and to take instant measurements at any time of day.

Limitations of the study

It is important to mention that the volunteers who participated in the study did not present any diagnosed heart disease (arrhythmias, tachycardia, bradycardia, etc.). Therefore, it is recommended that research be continued to consider the alterations that may occur to detect periods of hyperglycemia arising from any heart disease.

One of the main recommendations in the study carried out is to take into account demographic factors such as age, gender, and BMI, and alterations in additional characteristics to the ECG, such as QT interval, T wave, T interval, etc., that help improve the results provided in this research.

It is also important that to avoid errors in ECG signal sampling, it is necessary to follow a clinical measurement protocol, which includes resting for five minutes to stabilize HR and avoiding any sudden movement of electrodes or limbs that modify the ECG.

Future research should explore additional ECG features and incorporate larger datasets for improved accuracy. Further studies are needed to refine feature extraction techniques and validate findings in diverse populations.

This study demonstrates that ECG features, specifically HR and HRV, are useful for detecting periods of hyperglycemia with high accuracy. The importance of classifying people with normal BGLs and periods of hyperglycemia helps to eliminate errors in classifying people with DM. These errors are associated with people with very high BGLs who have not been clinically diagnosed with DM. Using only HR and HRV as input variables decreases the robustness of the models, and additional factors such as demographics or diseases mentioned above are necessary to improve classification.

In future work, a statistical analysis could also provide further insight into the relationship between the ECG variables analyzed and BGLs.

## Conclusions

This study demonstrates that ECG features, specifically HR and HRV, are useful in detecting hyperglycemic periods with high accuracy. The importance of the classification between people with normal BGLs and periods of hyperglycemia helps eliminate errors in the classification of people with DM. These errors are associated with people with very high BGLs who have not been clinically diagnosed with DM.

The results suggest that ECG analysis is an adequate adjunct for non-invasive glucose monitoring. ECG-based hyperglycemia detection offers a potential non-invasive alternative compared to traditional invasive methods. The study’s findings align with previous research that links HRV changes to glucose fluctuations.
